# MRI characterization of skeletal muscle size and fatty infiltration in long‐term trained and untrained individuals

**DOI:** 10.14814/phy2.15398

**Published:** 2022-07-19

**Authors:** Eric B. Emanuelsson, David B. Berry, Stefan M. Reitzner, Muhammad Arif, Adil Mardinoglu, Thomas Gustafsson, Samuel R. Ward, Carl Johan Sundberg, Mark A. Chapman

**Affiliations:** ^1^ Department of Physiology and Pharmacology Karolinska Institutet Stockholm Sweden; ^2^ Department of Nanoengineering University of California San Diego La Jolla California USA; ^3^ Department of Orthopaedic Surgery University of California San Diego La Jolla California USA; ^4^ Department of Radiology University of California San Diego La Jolla California USA; ^5^ Department for Women's and Children's Health Karolinska Institutet Stockholm Sweden; ^6^ Science for Life Laboratory KTH – Royal Institute of Technology Stockholm Sweden; ^7^ Centre for Host–Microbiome Interactions Faculty of Dentistry, Oral & Craniofacial Sciences, King's College London London UK; ^8^ Department of Laboratory Medicine Karolinska Institutet Huddinge Sweden; ^9^ Unit of Clinical Physiology Karolinska University Hospital Stockholm Sweden; ^10^ Department of Bioengineering University of California San Diego La Jolla California USA; ^11^ Department of Learning, Informatics, Management and Ethics Karolinska Institutet Stockholm Sweden; ^12^ Department of Integrated Engineering University of San Diego San Diego California USA

**Keywords:** adipose tissue, endurance training, human, resistance training

## Abstract

This study investigated body composition measures in highly trained and untrained individuals using whole‐body magnetic resonance imaging (MRI). Additionally, correlations between these measures and skeletal muscle gene expression were performed. Thirty‐six individuals were included: endurance‐trained males (ME, *n* = 8) and females (FE, *n* = 7), strength‐trained males (MS, *n* = 7), and untrained control males (MC, *n* = 8) and females (FC, *n* = 6). MRI scans were performed, and resting *M. vastus lateralis* (VL) biopsies were subjected to RNA sequencing. Liver fat fraction, visceral adipose tissue volume (VAT), total body fat, and total lean tissue were measured from MRI data. Additionally, cross‐sectional area (CSA) and fat signal fraction (FSF) were calculated from *Mm. pectoralis*, *M. erector spinae* and *M. multifidus* combined, *Mm. quadriceps*, and *Mm. triceps surae* (TS). Liver fat fraction, VAT, and total body fat relative to body weight were lower in ME and FE compared with corresponding controls. MS had a larger CSA across all four muscle groups and lower FSF in all muscles apart from TS compared with MC. ME had a lower FSF across all muscle groups and a larger CSA in all muscles except TS than MC. FE athletes showed a higher CSA in *Mm. pectoralis* and *Mm. quadriceps* and a lower CSA in TS than FC with no CSA differences found in the back muscles investigated. Surprisingly, the only difference in FSF between FE and FC was found in *Mm. pectoralis.* Lastly, correlations between VL gene expression and VL CSA as well as FSF showed that genes positively correlated with CSA revealed an enrichment of the oxidative phosphorylation and thermogenesis pathways, while the genes positively correlated with FSF showed significant enrichment of the spliceosome pathway. Although limited differences were found with training in females, our study suggests that both regular endurance and resistance training are useful in maintaining muscle mass, reducing adipose tissue deposits, and reducing muscle fat content in males.

## Graphical abstract

This study investigated body composition measures in highly trained and untrained individuals using whole‐body magnetic resonance imaging (MRI). Overall, this study suggests that both regular endurance and resistance training are, over time, appropriate to maintain muscle mass, reduce adipose tissue deposits, and reduce muscle fat content in male adults. While the results for female endurance athletes are mixed with regards to muscle CSA and FSF, significant reductions in body adipose tissue deposits and increased relative lean body tissue volume were detected. Lastly, to our knowledge, this is the first study correlating clinically measurable MRI parameters with gene expression data from RNA sequencing where we found several genes associated with m. vastus lateralis CSA and FSF.

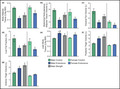

## 1 INTRODUCTION

Regular exercise training throughout life facilitates the preservation of skeletal muscle's ability to generate force, skeletal muscle mass, and aerobic capacity while reducing the risk of several diseases such as cardiovascular disease and type II diabetes (Chambers et al., 2020; Neufer et al., 2015; Zampieri et al., 2015). Additionally, lifelong exercise reduces the risk of developing various ailments such as low back pain (Kjaer et al., 2007; Neufer et al., 2015). In contrast to the positive effects of regular exercise training, physical inactivity and aging are associated with increased liver fat, visceral fat, decreased muscular strength, decreased skeletal muscle cross‐sectional area (CSA), and increased infiltration of adipose tissue into the muscle (Bertolotti et al., 2014; Biltz et al., 2020; Chambers et al., 2020; Manini et al., 2007). Importantly, there is evidence that both regular endurance and resistance training can reduce some of the detrimental effects of inactivity and aging on muscle (Chambers et al., 2020; Trappe et al., 2002; Zampieri et al., 2015). However, in any given previous study, the investigation into the effects of lifelong exercise on body composition is limited to isolated muscles groups, cohorts of mixed or one sex (i.e., male or female), specific adipose tissue deposits, and/or one exercise modality (i.e., aerobic‐ or resistance‐trained subjects). Given this, investigating body composition, various fat deposits, such as liver and visceral fat, and muscle morphology across the entire body in both males and females with a history of either lifelong aerobic or resistance exercise training will increase the understanding of how regular exercise serves to counteract physical inactivity and slow the aging process. Several reports have shown that physically inactive males and females over the age of 60 have a greater fat fraction in skeletal muscle than both young and elderly individuals who perform regular exercise training (Chambers et al., 2020; Csapo et al., 2014; Mikkelsen et al., 2017). Increased muscle fat content is associated with reduced force and power output per muscle unit and impaired insulin sensitivity (Boettcher et al., 2009; Delmonico et al., 2009; Konopka et al., 2018; Rahemi et al., 2015). However, endurance exercise training has been shown to decrease the fat fraction in muscle and increase power output in elderly sedentary individuals (Durheim et al., 2008; Lee et al., 2005; Murphy et al., 2012). In addition to endurance training, studies investigating the effect of resistance training on body composition have also shown improved muscular strength, increased lean mass, and decreased fat content in older adults, obese individuals, and people with various muscle wasting diseases (Long et al., 2021; Marcus et al., 2010; Nicklas et al., 2015; Waters et al., 2021). While previous studies mainly have examined the effects of exercise on performance related to blood measures (Contrepois et al., 2020) or muscle morphology of the thigh (Chambers et al., 2020; Trappe et al., 2002; Zampieri et al., 2015), investigations directly comparing fat fraction and muscle size in several skeletal muscle groups in long‐term trained and untrained individuals are lacking. By including several muscle groups, the understanding of how different muscles adapt to long‐term endurance or resistance training will be improved. Additionally, by studying visceral fat content as well as fat infiltration in central organs, such as the liver, further health benefits of regular exercise training and comparisons of different exercise regimens can be revealed.

Aside fromchanges in muscle morphology and body composition, it is well established that regular exercise alters skeletal muscle gene expression (Chapman et al., 2020; Lindholm et al., 2014; Stepto et al., 2009). Although these previous investigations provide valuable insights into muscle adaptation to exercise, invasive muscle biopsies are not clinically practical on a large scale. However, magnetic resonance imaging (MRI) is a routine clinical technique that allows for the noninvasive assessment of several parameters relevant to muscle health such as muscle CSA and fat content. Although some studies have characterized genes associated with muscle CSA and fat content (Mikkelsen et al., 2017; Sachs et al., 2019), to our knowledge, no previous study has examined how global gene expression is associated with clinically measurable MRI metrics of the local tissue. Understanding how gene expression correlates with parameters measured with MRI, such as fat content and muscle CSA, can begin to shed light on how clinically measured tissue‐level metrics are related to and can predict local gene expression. Thus, this association can provide candidate genes linked to clinically measurable parameters, such as CSA and fatty infiltration, in skeletal muscle.

Accordingly, the main aim of this study was to investigate body composition, body fat deposits, and muscle fat content and cross‐sectional area of skeletal muscles from four body compartments including nonweightbearing muscles (*Mm. pectoralis*), weightbearing muscles (*Mm. quadriceps*), and postural muscles (*M. erector spinae* and *M. multifidus* combined and *Mm. triceps surae*) in male and female individuals with different exercise training backgrounds. To understand how these clinical values are related to local muscle gene expression, a final aim was to associate MRI‐determined *M. vastus lateralis* fat content and CSA values with *M. vastus* *lateralis* global gene expression.

## 2 MATERIALS AND METHODS

### 2.1 Subjects

The study was approved by the Regional Ethical Review Board in Stockholm (application 2016/590‐31) and conducted in accordance with the Declaration of Helsinki. Written and verbal consent were both attained, and the subjects were informed that they may withdraw consent at any time during the experiment.

Thirty‐six middle‐aged individuals (males: *n* = 23; females: *n* = 13) were included in this investigation. Participants included: 1. endurance‐trained individuals (male endurance, ME; *n* = 8, female endurance, FE; *n* = 7) with at least 15 years of experience in regular endurance training, 2. resistance‐trained males (male strength, MS; *n* = 7) with at least 15 years of experience in regular resistance training, and 3. age‐matched untrained individuals (male control, MC; *n* = 8, female control, FC; *n* = 6) without any chronic disease and a body mass index (BMI) <26. A BMI cutoff of 26 was used for control subjects to make sure enhanced fat content would not be driven by the subjects being overweight or obese. No glucose tolerance test or assessment for insulin resistance (HOMA‐IR) was performed. A majority of the participants (*n* = 27) were fully characterized in a previous study (Chapman et al., 2020) investigating the effects of long‐term training on muscle gene expression, while six subjects (*n* = 2 MC, *n* = 4 MS) are from an unpublished study. All subjects went through physiological tests before inclusion, including a maximal oxygen uptake test (VO_2‐peak_) on a stationary bike and a unilateral maximal isokinetic knee extension test. To be included, endurance subjects had to have a VO_2‐peak_ above the 90th percentile for their age as defined by the LIV 2000 study (Ekblom‐Bak et al., 2011). Strength subjects needed to have a maximal torque output at least two standard deviations above the control group (i.e., >238.6 Nm). Additionally, all strength subjects were stronger than all endurance‐trained subjects and had a VO_2‐peak_ below the 90th percentile for their age. All trained subjects performed at least four exercise sessions per week. Control subjects had a VO_2‐peak_ below the 75th percentile for their age and perform exercise less than 2 times per week. Trained individuals with mixed endurance and resistance training backgrounds were excluded in order to isolate exercise modality‐specific adaptations. See Chapman et al. (2020) for a full methodological description of the physiological testing procedures. In brief, maximal knee extension torque performance was measured by an isokinetic knee extension at 90°/s using the Biodex Isokinetic System (System 4 pro, Biodex medical systems, New York, NY, USA). Three maximal knee extensions were performed on each leg, and the highest value of each leg was recorded. All subjects were familiarized with the machine and the testing procedure prior to performing the test. Measurements of aerobic capacity were performed on a bicycle ergometer by an incremental maximal oxygen uptake test. Time of day of the performance testing was not controlled for. Despite our recruitment efforts, we were unable to include resistance‐trained females with at least 15 years of resistance training history. Descriptive and physiological test statistics for all research participants are presented in Table 1.

**TABLE 1:** Descriptive statistics of subject characteristics

	Female		Male		
	Control	Endurance	Control	Endurance	Strength
*N*	6	7	8	8	7
Age (years)	43.8 (5.3)	42.3 (4.9)	42.4 (4.6)	41.6 (2.1)	41.0 (7.3)
Weight (kg)	65.9 (5.0)	56.4 (4.0)a	77.4 (11.9)	76.3 (5.7)	89.2 (10.7)^a^ ^,^ ^b^
Height (cm)	173 (7.9)	169.0 (3.9)	178.1 (10.1)	183.1 (5.6)	182.0 (8.5)
BMI (kg/m^2^)	22.6 (2.4)	20.1 (1.3)^a^	23.8 (1.4)	22.5 (2.9)	27.7 (3.2)^a^ ^,^ ^b^
VO_2_ peak (ml/kg·min)	31.8 (4.4)	57.1 (2.9)	36.0 (5.7)	62.9 (7.2)^a^ ^,^ ^c^	40.9 (4.6)
Strength output (Nm)	113.4 (18.9)	122.6 (8.9)	169.4 (34.6)	200.9 (26.4)	273.7 (34.8)^a^ ^,^ ^b^

*Note*: Baseline data are presented as mean (± standard deviation). Significance was set to *p*<0.05.

^a^ Significantly different from the control group.

^b^ Significantly different from the endurance group.

^c^ Significantly different from the strength group.

### 2.2 Magnetic resonance imaging

Full‐body MRI scans were performed on all research subjects. Prior to the scan, subjects were instructed to avoid any alcohol consumption and strenuous exercise training for 48 hours before the scan and avoid caffeine consumption on the day of the scan. Upon arrival at the scanning facility, participants rested in a supine position for 30 minutes before the MRI scan to minimize the influence of a posture‐related fluid shift on muscle volume (Berg et al., 1993). Images were obtained using a 1.5‐Tesla Siemens Magnetom Aera unit (Siemens Healthcare, Germany) as described previously (Lilja et al., 2018) and performed between 4 and 6 pm. A full‐body Dixon MRI pulse sequence was performed and continuous images were obtained from each subject with a voxel dimension of 2.2 × 2.2 × 3.5 mm (repetition time 6.69 ms; echo time 1: 2.23 ms, echo time 2: 4.77 ms; 224 × 168 matrix; field of view, 500 × 375 mm).

Measurements of body adipose tissue volume, lean body tissue volume, liver fat fraction, abdominal subcutaneous adipose tissue volume (ASAT), visceral adipose tissue volume (VAT), anterior thigh volume, and posterior thigh volume were performed as a service by AMRA medical (AMRA Medical AB, Linköping, Sweden) as previously described (Borga et al., 2015; Karlsson et al., 2015). In brief, VAT was defined as the adipose tissue within the abdominal cavity but excluding all subcutaneous adipose tissue. ASAT volume was quantified between the top of the femoral head to the top of the 9th thoracic vertebra (T9) (Borga et al., 2015). Relative body adipose tissue and lean body tissue were measured from the patella to the level of the T9 and divided by body mass (Borga et al., 2015; Karlsson et al., 2015).

### 2.3 Regions of interest—muscle size and fat content

To understand how long‐term exercise influences different muscle groups across the body, skeletal muscle regions of interest (ROIs) from five muscles or muscle groups of the upper body, lower back, upper leg, and lower leg were included for analysis. The muscles of interest included *Mm. pectoralis* (pectoralis), *M. erector spinae* and *M. multifidus* combined (ES/MF), *Mm. quadriceps* (quadriceps), *M. vastus lateralis* (VL), and *Mm. triceps surae* (TS). These muscle groups were selected in order to obtain a representative cross‐section of different muscle types—that is, weighbearing, nonweightbearing, and postural muscles. All muscles were segmented bilaterally by manual planimetry from one axial section using Horos v.3.3.6 (Horos, RRID:SCR_017340). One researcher, blinded from subject grouping, performed all analyses. In the upper body, ROIs were drawn at the maximal thickness of the *Mm. pectoralis*, which typically occurred at the T6 vertebral level. Skeletal muscle ROIs of ES/MF of the lower back were segmented at the mid‐L4 vertebral level (Berry et al., 2018). The measurement of the *Mm. quadriceps* and *M. vastus lateralis* was performed at the midpoint of the thigh (Chambers et al., 2020). The midpoint of the thigh was determined by measuring the length from the most lateral point of the trochanter major to the knee joint space (Delmonico et al., 2009). Finally, ROIs of TS were defined, where the maximal width of the lower leg was observed in the axial plane (Ruan et al., 2007).

### 2.4 Image analysis—muscle size and fat content

All ROIs were imported into a custom‐written MATLAB software program (Mathworks, Natick, MA) to measure CSA and fat signal fraction (FSF) (Berry et al., 2018). Images acquired using the Dixon MRI pulse sequence yield two sets of images, one where the signals from water and fat are perfectly in phase, and one where the water and fat signals are perfectly out of phase. A water‐only signal image (S_w_) was calculated by adding the in‐phase and out‐of‐phase images and dividing by 2. A fat‐only signal image (S_f_) can then be calculated by subtracting the water‐only signal image from the in‐phase image. From this, the independent contributions of both the water signal (S_w_) and the fat signal (S_f_) can be isolated within each voxel. The FSF was then calculated as follows:

$$\mathrm{Fat}\;\text{signal fraction}=\frac{S_{f}}{S_{f}+S_{w}}$$

### 2.5 Skeletal muscle quality

Skeletal muscle quality was determined as the maximal force output normalized to anterior thigh volume (N·m/cm^3^). The maximal force output was measured using a Biodex Isokinetic System (System 4 pro, Biodex medical systems, New York City, NY, USA) as previously described in Chapman et al. (2020).

### 2.6 Correlation between MRI and muscle transcriptomics data

To examine how fat content and CSA affect skeletal muscle health/metabolism at the gene level, we performed a direct comparison between these MRI metrics and skeletal muscle gene expression in the *M. vastus lateralis*. Cross‐sectional area and FSF analyses, at the midpoint of the thigh of VL, were correlated using Spearman's rank correlation coefficient, with skeletal muscle transcriptomic data of *M. vastus lateralis*, from the same individuals. The skeletal muscle transcriptome of a majority of the participants (*n* = 27) was fully characterized in a previous study (Chapman et al., 2020), whereas the remainder (*n* = 2 MC, *n* = 4 MS) are from an unpublished study in our lab. See Chapman et al. (2020) for a full description of the skeletal muscle biopsy procedure, RNA sequencing, the study design, and skeletal muscle transcriptomic data from all subjects in FE, FC, ME, three MS subjects, and six MC subjects. In brief, skeletal muscle biopsies were collected at rest, at least 72 h following the latest exercise session, from *M. vastus lateralis*. Muscle samples were immediately frozen in liquid nitrogen‐cooled isopentane and stored in −80°C until RNA isolation. Thirty milligrams of muscle sample were homogenized in 1.0 ml TRIzol reagent, and RNA was subsequently isolated using the standard TRIzol method as outlined in the manufactuer's protocol. RNA library preparation and sequencing were performed at the National Genomics Infrastructure, Sweden. All samples were then multiplexed in 1 lane and sequenced (2 × 50 bp paired‐end) on the Illumina NovaSeq 6000.

Pathway analysis of the significantly correlated genes was performed using the enrichr package in conjunction with the KEGG databases. Functional analyses were performed by using the enrichr GO Biological Process 2021 database and the PANTHER overrepresentation test for GO Biological Processes.

### 2.7 Statistical analysis

The sample size calculation was based on a previous investigation of whole thigh intermuscular adipose tissue in elderly healthy females with age‐matched lifelong endurance‐trained females (Chambers et al., 2020). Using α = 0.05, and a required power (1 − β) = 0.80, the desired sample size resulted in a sample size of at least six participants in each group.

All statistical analyses were conducted in GraphPad Prism 8 (GraphPad Prism, RRID:SCR_002798). Sex‐specific comparisons were performed using one‐way ANOVAs with Tukey's post hoc test for male subjects and an unpaired two‐tailed *t* test for the female group comparison. Due to the lack of a female strength‐trained group, sex comparisons in FSF could only be made between endurance and control subjects. Given this, we performed a two‐way ANOVA examining sex and training differences with endurance training. Sex differences in muscle volume and CSA were not investigated given the established sex differences that exist in muscle mass and muscle fiber CSA (Gallagher et al., 1997; Horwath et al., 2021). The relationship between maximal force output and anterior thigh volume was assessed with a simple linear regression. Throughout the entire study, statistical significance was set to a *p*‐value or an adjusted *p*‐value of ≤0.05, and all data are presented as mean ± standard error of the mean (SEM) unless indicated otherwise.

## 3 RESULTS

### 3.1 Body composition

**FIGURE 1:**
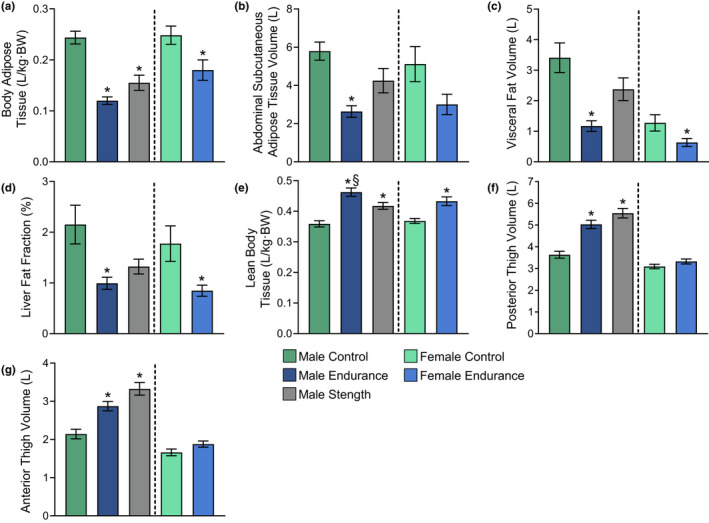
Baseline body composition characteristics of research subjects. (a) Adipose tissue volume relative to body mass, (b) abdominal subcutaneous adipose tissue volume, (c) visceral fat volume, (d) liver fat fraction, (e) lean body tissue relative to body mass, (f) posterior thigh volume, and (g) anterior thigh volume. Significance was set to 0.05. *Significantly different from the control group. ^#^Significantly different from the endurance group. §Significantly different from the strength group. All data are presented as mean ± SEM.

The untrained male and female control groups (MC and FC, respectively) had significantly more body adipose tissue relative to body mass compared with trained males (both ME and MS; *p* < 0.001) and females (FE; *p* = 0.028), respectively (Figure 1a). Additionally, FC and MC had significantly greater visceral fat volume and liver fat fraction than FE (*p* = 0.045 and 0.021) and ME (*p* = 0.001 and *p* = 0.009; Figure 1c,d). Also, MC had a greater ASAT volume compared with ME (*p* = 0.001), whereas no statistically significant difference was observed between FC and FE (*p* = 0.064; Figure 1b). Although MC had 30% more visceral fat and a 38% greater liver fat fraction compared with MS, no statistically significant differences were observed (*p* = 0.139 and *p* = 0.066, respectively; Figure 1c,d).

ME showed greater relative lean body tissue volume compared with both MC and MS (*p* ≤ 0.001 and 0.033, respectively), whereas MS had greater relative lean body tissue volume than MC (*p* = 0.005; Figure 1e). Similarly, FE displayed a higher relative lean body tissue volume compared with FC (*p* = 0.003; Figure 1e). MS and ME had significantly greater posterior (*p* < 0.001) and anterior (MS vs MC = *p* < 0.001, ME vs. MC = 0.003) thigh volumes compared with MC, whereas no significant differences were found between MS and ME (*p* = 0.163 and 0.079, respectively) or between FE and FC (*p* = 0.166 and 0.095, respectively; Figure 1f,g).

### 3.2 Fat signal fraction and cross‐sectional area—males

**FIGURE 2:**
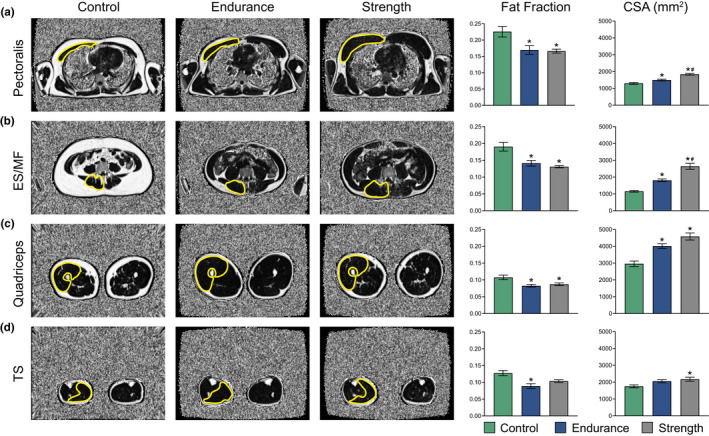
Fat signal fraction (fat fraction) and cross‐sectional area (CSA) in male subjects calculated from Dixon MRIs. Regions of interest of the corresponding muscle groups are highlighted in yellow. (a) *Mm. pectoralis*, (b) *M*. *erector spinae*and *M. multifidus* combined (ES/MF), (c) *Mm. quadriceps*, and (d) *Mm.* *triceps surae* (TS). All measurements were performed bilaterally, and the results were presented as averages of both left and right sides. Significance was set to 0.05. *Significantly different from the control group. ^#^Significantly different from the endurance group. All data are presented as mean ± SEM.

Endurance‐ and strength‐trained males had a significantly lower FSF in *Mm. pectoralis* (*p* = 0.004 and <0.001), ES/MF (*p* = 0.015 and 0.012), and *Mm. Quadriceps* (*p* = 0.006 and 0.031, respectively) compared with untrained control subjects (Figure 2a–c). ME had a significantly lower FSF compared with MC in TS (*p* = 0.001), whereas no statistically significant differences were observed between MC and MS (*p* = 0.053) or MS and ME (*p* = 0.284; Figure 2d) in TS. Additionally, ME and MS had significantly larger muscle CSA of *Mm. pectoralis* (*p* = 0.001 and <0.001), ES/MF (*p* = 0.038 and <0.001), and *Mm. quadriceps* (*p* = 0.001 and <0.001, respectively) than MC (Figure 2a–c). Furthermore, MS showed larger muscle CSA of both *Mm. pectoralis* and ES/MF compared with ME (*p* < 0.001 and *p* = 0.001; Figure 2a,b). Finally, MS had larger TS CSA than MC (*p* = 0.019), whereas no statistically significant differences were observed in TS between MS and ME or ME and MC (Figure 2d).

### 3.3 Fat signal fraction and cross‐sectional area—females

**FIGURE 3:**
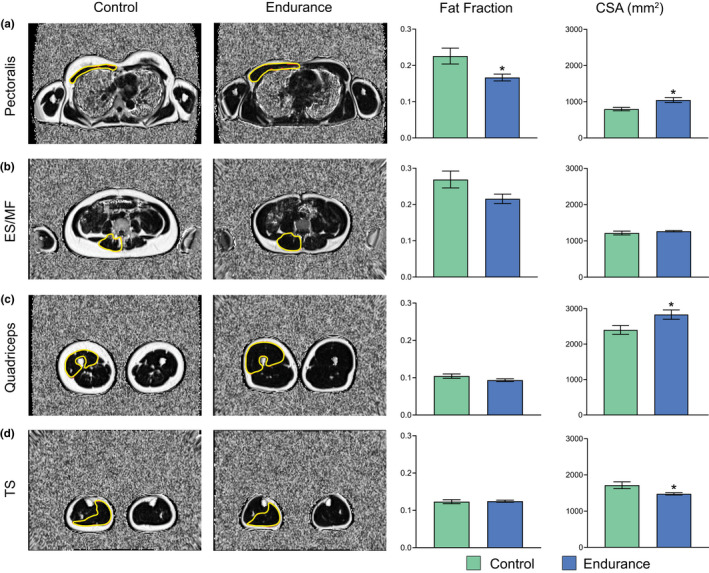
Fat signal fraction (fat fraction) and cross‐sectional area (CSA) in female subjects calculated from Dixon MRIs. Regions of interest of the corresponding muscle groups are highlighted in yellow. (a) *Mm p ectoralis*, (b) *M.* *erector spinae* and *M.* *multifidus*combined (ES/MF), (c) *Mm.* quadriceps, and (d) *Mm.* triceps surae (TS). All measurements were performed bilaterally, and the results were presented as averages of both left and right sides. Significance was set to 0.05.*Significantly different compared with the control group. All data are presented as mean ± SEM.

Figure 3 depicts the comparisons of skeletal muscle CSA and FSF between the endurance‐trained and untrained female subjects. FE has a significantly lower FSF in *Mm. pectoralis* compared with FC (*p* = 0.024; Figure 3a). Although a 20% lower FSF fraction was observed in the lower back muscles of FE compared with FC (*p* = 0.062), no muscle groups other than *Mm. pectoralis* were significantly different in FSF between the groups (Figure 3b–d). *Mm. pectoralis* and *Mm. quadriceps* CSA were larger in FE compared with FC (*p* = 0.013 and 0.038; Figure 3a,c), whereas FC showed a larger TS CSA compared with the endurance group (*p* = 0.026; Figure 3d). Finally, no difference was seen between the two female groups in the lower back muscle compartment (ES/MS) CSA (*p* = 0.339; Figure 3b).

### 3.4 Fat signal fraction—sex differences

The only statistically significant FSF difference detected between ME and FE was found in TS (*p* = 0.002), whereas no sex differences were detected in FSF across any muscle group between MC and FC.

### 3.5 Muscle quality

**FIGURE 4:**
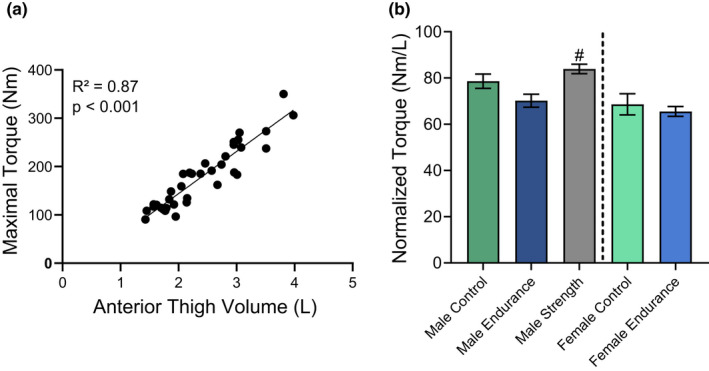
Relationship between quadriceps torque and anterior thigh volume. (a) Pooled relationship between torque output and anterior thigh volume of all test subjects, (b) torque output (N·m) per anterior thigh volume (L). Significance was set to 0.05. ^#^Significantly different from the endurance group. All data are presented as mean ± SEM.

In order to understand how muscle performance relates to muscle structure, maximal torque production was correlated with anterior thigh volume. A significant correlation (*R* ^2^ = 0.87, *p* < 0.001) was observed between maximal torque and anterior thigh volume when the study population was pooled (Figure 4a). However, when the torque data were normalized by thigh volume, the only significant difference in normalized torque was found between MS and ME where the normalized torque output was 19.5% higher in MS compared with ME (*p* = 0.007; Figure 4b).

### 3.6 Correlation between FSF and CSA with skeletal muscle gene expression

**FIGURE 5:**
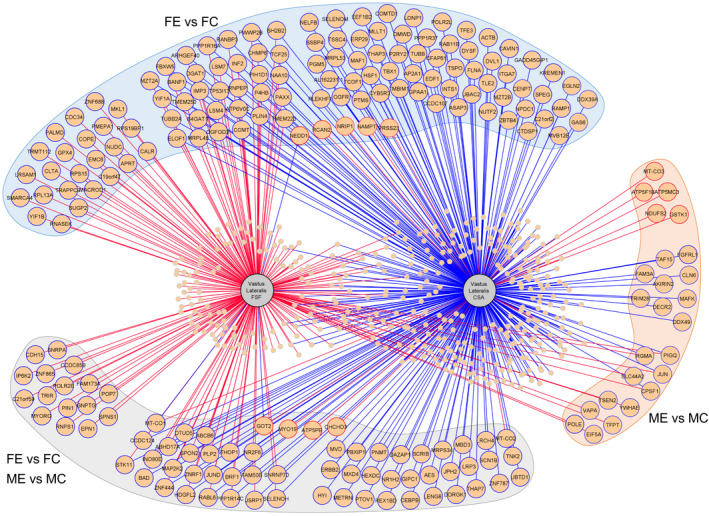
Network of all significantly (adjusted *p*‐value <0.05) correlated genes (peach circles) with *M. vastus lateralis* cross‐sectional area (CSA) and a fat signal fraction (FSF). Large peach circles represent the genes differentially expressed between the highlighted group comparison (FE vs. FC, ME vs. MC, and FE vs. FC/ME vs. MC combined). Small peach circles represent genes differentially expressed in nonhighlighted comparisons (MS vs. MC, ME vs. MS, FE vs. ME, and FC vs. MC) or genes that are not differentially expressed between groups (see Table S1 for a full list of genes and correlations). Line color indicates the nature of the correlation: blue, negative correlation; red, positive correlation. The circle border color indicates the direction of the gene expression in the highlighted comparison: blue, downregulated; red, upregulated. FC, female control; FE, female endurance; MC, male control; ME, male endurance; and MS, male strength.

In order to characterize how certain muscle parameters are associated with the expression of genes in *M. vastus lateralis*, we correlated VL CSA and FSF with global gene expression measured from muscle biopsies (from Chapman et al. (2020)) from the same subjects (Figure 5). Genes with a statistically significant (adjusted *p*‐value <0.05) positive or negative correlation with VL CSA (28 positively correlated genes, Spearman's rank correlation coefficient range: 0.63–0.47; 415 negatively correlated genes, Spearman's rank correlation coefficient range: −0.75 to −0.47) and FSF (242 positively correlated genes, Spearman's rank correlation coefficient range: 0.61–0.47; 3 negatively correlated genes, Spearman's rank correlation coefficient range: −0.53 to −0.48) were identified and are presented in Table S1. A large portion of the significantly correlated genes (219/587) were found to be differentially expressed with endurance training (Figure 5), whereas only one correlating gene was differentially expressed between MS and MC (Table S1). Between the sexes, 38 of the significantly correlated genes were differentially expressed between the male and female subjects—9 genes in common between both FE vs. ME and FC vs. MC, 26 genes between FC vs. MC, and 3 genes between FE vs. ME. A total of 339 genes were significantly correlated with CSA and FSF but were not found to be differentially expressed between any of the experimental groups (Figure 5). Functional analysis revealed that genes positively correlating with CSA are significantly related to oxidative phosphorylation, thermogenesis, and mitochondrial structure. Functional analysis of genes positively correlated with FSF and negatively correlated with CSA revealed a significant relationship with functions related to mRNA processing. The spliceosome pathway was significantly enriched among the genes positively correlated with FSF. As only three genes were negatively correlated with FSF, no pathway or functional analysis was performed.

## 4 DISCUSSION

Overall, this study suggests that both regular endurance and resistance training are, over time, appropriate to maintain muscle mass, reduce adipose tissue deposits, and reduce muscle fat content in male adults. Although the results for female endurance athletes are mixed with regards to muscle CSA and FSF, significant reductions in body adipose tissue deposits and increased relative lean body tissue volume were detected. Lastly, to our knowledge, this is the first study correlating clinically measurable MRI parameters with gene expression data from RNA sequencing, where we found several genes associated with *M. vastus lateralis* CSA and FSF.

Both endurance and resistance‐trained males generally had less skeletal muscle fat content and larger CSA compared with untrained males in both upper and lower body muscle groups. These findings are in line with previous studies that showed similar results in the thigh muscles in lifelong endurance‐trained subjects (Chambers et al., 2020; Mikkelsen et al., 2017) and decreased fat infiltration in the *M. multifidus* of 40‐year‐old persons active in sports compared with less‐active adults (Kjaer et al., 2007). Unlike endurance‐trained males, where muscle fat content was significantly reduced in all muscle groups compared with controls, the female endurance group had a similar skeletal muscle fat fraction compared with untrained females in three out of the four muscles measured. The discrepancy between our findings in the male and female cohorts could be explained by the fact that previous research has shown that males have an increase in fat infiltration in the lower back muscles beginning in their 30s, while this increase does not occur in females until their 50–60s (Crawford et al., 2016). Since our research subjects were between the ages of 34–53, we hypothesize that exercise training in males has prevented this age‐related increase in muscle fat content, described by Crawford et al, that typically begins in the third decade of life in males. However, since all of our female subjects were under 50 years old, this age‐related increase in muscle fat content has yet to occur, resulting in limited differences in muscle fat content between the trained and untrained female subjects. Indeed, when investigating elderly (70–80 years old) female endurance athletes, Chambers et al. (2020) showed a decrease in whole thigh muscle fat content compared with sedentary controls. This same pattern, however, was not seen in TS where both endurance‐trained and sedentary elderly females had similar muscle fat content. Based on these previous studies, we speculate that female athletes who continue an endurance training regimen into older age will have lower muscle fat content compared with their sedentary counterparts. Another explanation for the differences between FE and ME could be influenced by aerobic capacity, as ME had a VO_2_‐ peak 11.5% higher than FE (62.9 and 56.4 ml·kg^−1^·min^−1^, respectively). Indeed, Chambers et al. (2020) have previously shown that endurance‐trained individuals with higher VO_2max_ have a lower skeletal muscle fat fraction than recreationally endurance‐trained individuals (Chambers et al., 2020). Thus, our data in the context of the existing literature demonstrate that regular exercise training throughout life helps to maintain a low skeletal muscle fat fraction.

Increased levels of skeletal muscle fat content can impair metabolic function since it has been shown to be negatively correlated with insulin sensitivity (Boettcher et al., 2009). Furthermore, Sachs et al. (2019) proposed that intermuscular adipose tissue can negatively regulate skeletal muscle insulin sensitivity, possibly by secreting free fatty acids and cytokines which might modify the extracellular matrix and promote local inflammation. In addition to impaired muscle insulin sensitivity, increased muscle fat content has also been positively correlated with increased protein expression of myostatin, a well‐established inhibitor of skeletal muscle growth (Konopka et al., 2018), which emphasizes the importance of regular exercise in maintaining metabolic and muscular function. Additionally, decreased muscle CSA and increased fat content in the lumbar paravertebral muscles have been strongly related to lower back pain in adult men and women (Kjaer et al., 2007; Parkkola et al., 1993; Shahidi et al., 2017). Considering the higher fat content in untrained males across multiple muscle groups in this study, it may be suggested that therapeutic interventions should target whole‐body resistance and/or endurance exercise to reduce skeletal muscle fat fraction and, thus, improve skeletal muscle insulin sensitivity, decrease low back pain, and increase muscle CSA (Parkkola et al., 1993; Schumann et al., 2022; Waters et al., 2022).

To get a better understanding of the potential links between muscle fat content, CSA, and muscle function, we performed functional analysis on the genes expressed in the *M. vastus lateralis* that significantly correlated with *M. vastus lateralis* fat content and CSA. Genes positively correlated with larger *M. vastus lateralis* CSA were significantly associated with the oxidative phosphorylation and thermogenesis pathways. Additionally, the GO biological process database revealed a significant relationship between larger CSA and cristae formation and inner mitochondrial membrane organization, likely driven by the endurance groups. It is well established that endurance training leads to increased mitochondrial biogenesis, improved oxidative phosphorylation, increased activity of several mitochondrial enzymes, increased inner mitochondrial membrane surface area, and increased mitochondrial cristae density (Granata et al., 2021; Nielsen et al., 2017). Thus, since physical inactivity and a sedentary lifestyle are associated with a reduction of the expression of genes involved in mitochondrial processes and ATP production (Pillon et al., 2020), the link between increased CSA and oxidative phosphorylation and mitochondrial structure can be explained by the combination of the high aerobic capacity of the endurance group and a reduced expression of mitochondrial genes in the control groups.

Among the significantly enriched functions from genes positively correlating with *M. vastus lateralis* fat content and negatively correlated with CSA, several processes related to RNA processing were found. Specifically, RNA splicing and the spliceosome pathway were significantly enriched with higher fat content. The spliceosome forms functional complexes to regulate alternative splicing of pre‐mRNAs in muscle (Papasaikas & Valcárcel, 2016). Previous work by Ubaida‐Mohen et al. found that the main spliceosome complex proteins increase with aging in skeletal muscle and that almost 5000 protein‐coding transcripts were alternatively spliced with increasing age (Ubaida‐Mohien, Lyashkov, et al., 2019). Functional analysis of these alternatively spliced genes demonstrated that many were related to mitochondrial proteins (Ubaida‐Mohien, Lyashkov, et al., 2019). Ubaida‐Mohen et al. go on to discuss that these age‐associated changes in skeletal muscle gene splicing occur to stave off energetic deficiencies that develop with aging (Ubaida‐Mohien, Lyashkov, et al., 2019). Additionally, higher self‐reported physical activity has been related to a downregulation of proteins involved in the splicing machinery in muscle (Ubaida‐Mohien, Gonzalez‐Freire, et al., 2019). Thus, taken together with these previous studies, our data suggest that regular exercise training and the corresponding reduction in muscle fat content can be one factor, among several, which helps to preserve a “younger” muscle phenotype by hampering age‐related alternative splicing of skeletal muscle genes. Furthermore, our data demonstrate that clinically measurable parameters such as CSA and fat content can be indicators of the overall metabolic health of the muscle.

The fact that MS displayed larger CSA of *M. pectoralis* and the lower back muscles than both MC and ME was expected as these muscles are highly activated in common resistance training exercises such as bench press and deadlifts (Ferland & Comtois, 2019). Additionally, the fact that MS and both endurance groups had larger *M. quadriceps* CSA than the control groups is in accordance with previous literature (Chambers et al., 2020; Chapman et al., 2020; Ubaida‐Mohien et al., 2022). However, not only was a larger CSA found in the weightbearing muscles of the thigh in trained individuals but also in a nonweightbearing muscle (i.e., the *M. pectoralis*, Figures 2a and 3a), despite the lack of regular resistance exercise reported in the weekly training routine of ME and FE. The larger *M. pectoralis* CSA could be the result of enhanced ventilation during endurance exercise and also the activation of the pectoralis muscle during running (Aslan et al., 2019; Milligan et al., 2014). Surprisingly, the untrained females had larger TS CSA than FE. Since TS is frequently activated during regular walking, the measured CSA difference could be explained by the fact that FC individuals were 15% heavier than FE, and thus TS carries a heavier load in FC than FE (Ericson et al., 1986). Despite the TS CSA difference in female participants, the CSA of all other muscle groups investigated were larger or equal in the trained groups compared with the corresponding control group. Thus, our data demonstrate that both regular resistance and endurance exercise can maintain and/or enhance skeletal muscle mass compared with untrained individuals.

Previous research has shown equivocal results on the relationship between skeletal muscle CSA, fatty infiltration, and muscle contractile performance (Chambers et al., 2020; Delmonico et al., 2009; Konopka et al., 2018). In the current study, a significant difference in normalized quadriceps torque (torque per anterior thigh volume) was only detected between MS and ME. The difference in normalized torque shown between MS and ME is unsurprising considering the different nature of the two exercise modalities and the different muscle fiber composition between groups (Chapman et al., 2020; Egan & Zierath, 2013). When examining the control groups' normalized torque, no significant differences were found between the control group and any trained group which agrees with recent data from Chambers et al. (2020). However, a study by Konopka et al. (2018) showed an inverse correlation (*r* = −0.67) between intermuscular adipose tissue and normalized peak power (Watts/muscle CSA). This discrepancy from the current study could be explained by the large age range investigated in Konopka et al. (2018). Specifically, both younger (average age 20) and older adults (average age 69 and 74) were included, which could explain their findings considering the established positive correlation between muscle fat content, declining power output, and age (Crawford et al., 2016).

Body composition differed significantly between groups depending on exercise background. The untrained male and female subjects had significantly more adipose tissue and significantly less lean tissue relative to body weight compared with both the endurance‐ and the resistance‐trained subjects. Furthermore, FC and MC had more visceral fat, ASAT, and a greater percentage of liver fat than FE and ME. Increased fat content in control subjects compared with endurance subjects was expected considering the lower body fat percentage typically seen in both younger endurance athletes and in masters' athletes (Fleck, 1983; Piasecki et al., 2019; Ubaida‐Mohien et al., 2022). Interestingly, no differences in fat volume were seen between ME and MS, although endurance athletes typically have a lower total body fat percentage compared with strength athletes (Yeater et al., 1996). Together, these findings further highlight the importance of regular exercise to maintain metabolic health as excess visceral fat and liver fat have been related to metabolic disease and impaired insulin sensitivity (Fabbrini et al., 2009; Gan et al., 2003). Finally, although the untrained individuals had a significantly higher liver fat percentage than endurance‐trained athletes, the liver fat percentage displayed in the control groups is still in the normal healthy range (Szczepaniak et al., 2005).

The cross‐sectional design of the current study has some limitations, which primarily center around the difficulty of drawing causal inferences between exercise history and the measured parameters. A cross‐sectional study is only a snapshot of the current situation, and the detected group differences could arise for reasons other than exercise training—for example, due to genetic or dietary differences between our subjects. However, previous longitudinal studies have shown increased fat infiltration and decreased muscle strength with aging (Delmonico et al., 2009) and that regular physical activity can counteract these negative effects in knee extensors (Goodpaster et al., 2008). Although cross‐sectional studies cannot definitively infer causality, based on previous longitudinal work (Delmonico et al., 2009; Goodpaster et al., 2008), we herein assume that group differences detected in the current study are caused by the differences in exercise background. Additionally, for ethical and monetary reasons, it is not feasible to perform an exercise training study over 15 years which would dictate whether or not a study participant exercises. Another limitation of the current study is the lack of resistance‐trained female athletes, which we were unable to recruit for participation. This limitation means that we are unable to make any conclusions on how females adapt to long‐term resistance training, and claims on how long‐term resistance training influences skeletal muscle in males should not be extrapolated to females. Lastly, it should be noted that the only force measurement performed was an isokinetic knee extension involving *M. quadriceps femoris* and therefore no conclusions related to muscle function should be extrapolated to the other muscles studied (TS, ES/MF, and *M. pectoralis*). Future investigations should test the muscle function in different muscle groups, such as nonweightbearing upper body muscles, to further understand muscle quality in trained and untrained individuals.

In conclusion, we show that both regular endurance and resistance exercise are, independently, over time, sufficient to maintain skeletal muscle mass, and reduce the infiltration of adipose tissue in adults. These findings further support the notion that regular exercise training should be incorporated into various therapeutic interventions, where the goal is not only to maintain or increase muscle mass but also to reduce the levels of fat content in skeletal muscle, which can improve metabolic health by increasing oxidative capacity, mitochondrial function, and reducing alternative splicing. While we show that both endurance training and resistance training independently seem efficient for this purpose, the combination of the two training modalities, in a whole‐body exercise routine, could add complementary additional health benefits by improving aerobic capacity, muscular strength, and maintaining a reduced skeletal muscle fat content.

## AUTHOR CONTRIBUTIONS

E.B.E., D.B.B., T.G., C.J.S., and M.A.C. conceived of the study design, M.A.C. and S.M.R. recruited and screened research subjects. E.B.E., D.B.B., and M.A. analyzed MRI scans, processed data, and performed the statistical analysis. E.B.E., D.B.B., M.A., S.M.R., T.G, S.R.W, C.J.S., A.M., and M.A.C. were all involved in data interpretation. E.B.E and M.A.C prepared figures. E.B.E. and M.A.C. wrote the paper and all authors were involved in editing the paper.

## FUNDING INFORMATION

This work was financially supported by grants from the Swedish Research Council (2018‐02932) and the Swedish Center for Sports Research (P2017‐0163, P2017‐0023, and D2018‐0007). M.A.C. was supported as a postdoctoral scholar through the Whitaker International Program. S.M.R. was supported by a PhD training grant from Karolinska Institute.

## CONFLICT OF INTEREST

No conflict of interest, financial or otherwise, are declared by the authors.

## Supporting information




Table S1
